# Using a Clinical Workflow Analysis to Enhance eHealth Implementation Planning: Tutorial and Case Study

**DOI:** 10.2196/18534

**Published:** 2021-03-31

**Authors:** Stephanie Staras, Justin S Tauscher, Natalie Rich, Esaa Samarah, Lindsay A Thompson, Michelle M Vinson, Michael J Muszynski, Elizabeth A Shenkman

**Affiliations:** 1 Department of Health Outcomes and Biomedical Informatics College of Medicine University of Florida Gainesville, FL United States; 2 Institute for Child Health Policy University of Florida Gainesville, FL United States; 3 Behavioral Research in Technology and Engineering Center Department of Psychiatry University of Washington Seattle, WA United States; 4 Department of Radiology College of Medicine University of Florida Gainesville, FL United States; 5 College of Social Work Florida State University Tallahassee, FL United States; 6 Department of Pediatrics College of Medicine University of Florida Gainesville, FL United States; 7 Department of Clinical Sciences Florida State University College of Medicine Orlando Regional Campus Orlando, FL United States

**Keywords:** workflow, implementation science, primary care, eHealth, stakeholder engagement

## Abstract

eHealth apps often fail to improve clinical outcomes due to poor integration with clinical workflow—the sequence and personnel needed to undertake a series of tasks for clinical care. Our central thesis is that eHealth interventions will be more effective if the clinical workflow is studied and taken into consideration for intervention implementation. This paper aims to provide an introductory tutorial on when and how to use a clinical workflow analysis to guide the implementation of eHealth interventions. The tutorial includes a step-by-step guide to conducting a clinical workflow analysis in planning for eHealth implementation. We began with a description of why a clinical workflow analysis is best completed before the implementation of eHealth interventions. Next, we described 4 steps needed to perform the clinical workflow analysis: the identification of discrete workflow components, workflow assessment, triangulation, and the stakeholder proposal of intervention implementation. Finally, we presented a case study of a clinical workflow analysis, which was conducted during patient visits of patients aged 11 or 12 years from 4 diverse pediatric or family medicine clinics to plan the implementation of a tablet-based app for adolescent vaccination. Investigators planning the implementation of new eHealth interventions in health care settings can use the presented steps to assess clinical workflow, thereby maximizing the match of their intervention with the clinical workflow. Conducting a prospective workflow study allows for evidence-based planning, identifying potential pitfalls, and increasing stakeholder buy-in and engagement. This tutorial should aid investigators in increasing the successful implementation of eHealth interventions.

## Introduction

### Background

Within health care settings, one of the most common reasons eHealth apps fail to effectively increase the health outcomes they are designed to aid and improve quality of care is incompatibility between the app and clinical workflow [1-7]—the series of tasks conducted to complete clinical care in what order and by whom. Incompatibilities often stem from a wait-and-see method of implementation common to many eHealth interventions whereby an intervention is introduced into a clinical setting and the clinical workflow either adjusts to accommodate or does not [8,9]. Frequently, a lack of integration with clinical workflow results in clinical staff creating work-arounds or adaptations that interfere with the core components of the intervention [2-4,10]. For example, nurses reported checking boxes on forms that were not applicable to move the screens forward and others reported supplementing the electronic records with handwritten notes on paper [10]. Many eHealth apps would be more effective if a tailored implementation plan was created based on a systematic clinical workflow analysis performed before implementation [11].

Several methods for assessing clinical workflow are available [12-17]. Most notably, the Agency for Healthcare Research and Quality (AHRQ) has a digital toolkit of resources to guide investigators in studying clinical workflow [12]. In addition, Ozkaynak et al [13] have written an overview chapter of clinical workflow, including definitions of workflow, several workflow evaluation approaches, and examples of visualizing workflow [13]. Other studies describe select methods of studying clinical workflow, including electronic health record (EHR) audit logs, direct observation by trained external staff, clinic staff reporting, and sensor-based recordings [14-17]. Despite evidence that an intervention’s compatibility with the local setting is pertinent to success [18], there is limited information clearly describing how to increase compatibility with clinical workflow before implementation within the eHealth literature.

According to the Consolidated Framework for Implementation Research (CFIR), a well-known implementation research framework, interventions are not inherently compatible with specific settings, and adaptation is required to maximize success [19,20]. Following this meta-theory, interventions can be adapted as long as the changes occur within the adaptable periphery—components that do not compromise the intervention’s core components essential for the affect. One type of adaptation to improve user compatibility, a key construct from the diffusion of innovations theory incorporated within the CFIR inner setting [19,21,22] that can frequently be made without affecting the intervention’s core components, is integration with the clinic’s workflow. To ensure that adaptations enhance compatibility and remain within the adaptable periphery, it is suggested that the investigators and clinic staff collaborate to develop adaptations. In addition to engaging the clinical staff stakeholders, a key construct of CFIR and a critical component of integrating eHealth into primary care, this collaboration increases the likelihood of meeting two additional constructs within the CFIR process domain: identifying clinical champions and garnering the support of opinion leaders [19,22,23].

### Objectives

This paper offers a simple-to-follow methodology for studying clinical workflow and planning for implementation with clinic staff. It provides tools adapted from previously published strategies to aid researchers in identifying clinic-specific adaptations that will increase the compatibility of eHealth interventions with clinical workflow and thus improve outcomes [24,25]. We present 4 steps for assessing clinical workflow and improving intervention compatibility with clinical settings, and a case study illustrating these steps. First, identify which components of the intervention are critical and when these components need to occur during a clinic visit. Second, choose from a variety of described methods to observe the existing clinical workflow relevant to the intervention. Third, confirm their findings using a second workflow assessment strategy. Finally, consult the clinic staff on the best ways to adapt the intervention compatibility with the confirmed clinical workflow. The purpose of this paper is to provide a tutorial for eHealth researchers on how to assess clinical workflow and use the knowledge gained to adapt interventions for maximal implementation success.

## Assessing Clinical Workflow

### Workflow Assessment Steps

Clinical workflow assessments for implementation planning can follow 4 steps (Table 1): (1) the identification of discrete workflow components, (2) workflow assessment, (3) triangulation, and (4) the stakeholder proposal of intervention implementation.

**Table 1:** Steps for conducting a workflow assessment.

Step	Purpose	Methods	Example tools
Identify discrete workflow components	Define what is necessary to make the intervention work	Select locations, interactions, and tasks	Review direct observation checklist (Multimedia Appendix 1) and other checklists to select clinical practices [26]
Workflow assessment	Create a model of the clinical workflow	Direct observation, interviews, sensor-based investigations, EHR^a^ audit logs, and job task diaries	Review direct observation form (Multimedia Appendix 1)
Triangulation	Confirm rigor of clinical workflow model	Direct observation, interviews, sensor-based investigations, EHR audit logs, and job task diaries	Review semistructured template (Multimedia Appendix 2)
Stakeholder proposal	Plan intervention implementation based on stakeholder preferences	Interviews	Review semistructured template (Multimedia Appendix 2)

^a^EHR: electronic health record.

### Step 1: Identification of Discrete Workflow Components

The first step in conducting a clinical workflow analysis for improving eHealth implementation planning is to identify the discrete components of the clinical workflow that need to be measured. Owing to the multitude of tasks occurring during clinical visits, it is imperative to clearly define what activities researchers should track to ensure that each is collected and documented consistently. Tracked activities may fall under 3 observable workflow categories: location, interactions, and tasks. Location refers to where and how long individuals are physically present in specific areas of a clinic. Interactions include face-to-face conversations and moments in which health records or patients transition from one provider to another. Tasks include a review of health records, measures collected (eg, weight, blood pressure), interventions administered (eg, giving prescription or vaccine), and administrative functions (eg, patient check-in or scheduling). For each task considered, the actors (patient, provider, or support staff) may vary.

To identify the discrete workflow components for the intervention, consider what is necessary to make the intervention work as intended in the setting. Imagine how the intervention will be integrated into the clinic and considered the following questions: At what time during a clinic visit is the most useful for the patient and provider to access the intervention? What specific tasks need to occur to have the intervention in the hands of those who need it at the right time during the clinical encounter? What types of clinical situations require alternative planning? When choosing the discrete workflow components of interest, it is important to consider the minimum necessary rule and evaluate tasks directly relevant to an intervention’s implementation that do not compromise patient privacy or impede care delivery.

### Step 2: Workflow Assessment

A second step in conducting a workflow analysis to improve eHealth intervention implementation is to assess the clinical workflow. Several strategies can be used to assess clinical workflow, including direct observation, clinical staff reporting, interviews with staff or patients, sensor-based investigations, EHR audit logs, and job task diaries [14,16,27-31]. We focused on direct observation by a member of the research staff because this method offers advantages over other possible choices, including an in-depth analysis of complex interactions and clinical work-arounds, limited equipment and technology needs, and the potential to increase clinical staff engagement [5,16,29].

Workflow assessment via direct observation can occur in 3 steps: (1) identify who you wish to observe, (2) complete informed consent with potential participants, and (3) directly observe the workflow using standardized data collection tools to systematically record observations. Before direct observation, engage with clinic leadership to ensure that the observed activities will not affect medical staff employment. Before the observation days, explain the informed consent to the identified staff so that they can thoughtfully consider participation. Observations should be scheduled for clinic days in which relevant patients have scheduled visits. Once patients express interest to clinical staff, patient consent can be obtained. Care must be taken to avoid disrupting the standard clinical flow to maintain high-quality research and clinical care. Although the number of observed patients can be influenced by practical considerations (eg, travel expenses and clinical receptiveness), to achieve a valid sample, patients should be observed until additional observations no longer present new information (ie, saturation is achieved) [32,33].

The use of a standardized observation form increases the rigor and reproducibility of workflow observations and can be incorporated into any workflow assessment method chosen [15]. The standardized observation form should include a simple method to record the location and primary actors for each of the discrete workflow components of interest. Multiple data collection forms and observers may be necessary because clinics have several actors attending to each patient simultaneously. Examples of standardized observation forms, including an adaptable Microsoft Access database, can be found in the AHRQ health information technology workflow assessment toolkit [34]. Including timestamps of observed activities in the standardized observation form enables researchers to conduct what is considered the gold standard measurement for clinical workflow assessment, a time and motion study [29]. Time and motion studies evaluate the activities and duration of each activity of the clinical workflow.

### Step 3: Triangulation

A third step in conducting a clinical workflow analysis for improving eHealth intervention implementation is method triangulation: the verification of findings from a different viewpoint by answering similar questions with a different method [35]. Triangulation is a common strategy for enhancing the rigor of qualitative studies [32]. If triangulation reveals small deviations, the workflow should be updated. If triangulation reveals large deviations, additional data collection should be considered.

All the options mentioned for primary workflow assessment are possible methods for triangulation [16,27-31]. An additional option for triangulation is member checking of results, whereby the observed workflow is presented visually to the stakeholders via a sequential task analysis, line graphs, flow charts, and diagrams [13,17,36,37]. The AHRQ provides several examples of how to present workflow observations as flowcharts [38].

### Step 4: Stakeholder Proposal of Intervention Implementation

It is useful for stakeholders or actual clinical users to evaluate how the intervention will be compatible with their clinical workflow. Stakeholder opinions on implementation can be obtained during the triangulation phase through interviews or planning meetings. The planning process should follow the community participatory principles of engagement and participation so that the clinic staff feel that their insights and expertise are critical to the project [39]. Examples of critical research staff attitudes include mutual respect and genuineness, transparent processes, and balancing power [39]. Critical components for the success of stakeholder engagement include genuine partnerships, strategic selection of clinicians at each site to be involved in the project, and accommodation of stakeholder needs (eg, patient care responsibilities) [40]. A framework for operationalizing the engagement of partners, including examples of practices from research projects, is available from the Patient-Centered Outcomes Research Institute [41]. Finally, research staff should use this opportunity to discover or gain understanding into further adaptation needed for special situations (eg, acute visits vs wellness visits).

## Case Study

### Overview: Protect Me 4

We developed a tablet-based eHealth app for primary care clinics called *Protect Me 4*. The app is intended for use by parents of adolescents at the beginning of their child’s appointment to help parents learn about recommended vaccines and explore educational information addressing common hesitations. At the time of the appointment, the app also prompts providers of due vaccines and parents’ selected hesitations to prepare the provider to lead more efficient and effective vaccine discussions.

Our pilot trial demonstrated that the intervention successfully increased initiation of our main target, human papillomavirus (HPV) vaccine [6]. The app’s overall effectiveness was limited because only 8% (57/1062) of the eligible families used our app. On the basis of poststudy interviews with clinic staff, we hypothesized that the clinic workflow was not aligned with the implementation strategy. Thus, before our second study with an enhanced version of the app, we conducted a workflow analysis at 4 participating primary care clinics to understand how we could adapt our intervention to improve compatibility. All activities were approved by the University of Florida Institutional Review Board.

### Step 1: Identification of Discrete Workflow Components

We identified 3 workflow-related conditions necessary for the success of the intervention. First, a parent needed 5-10 minutes to complete the application before the child saw the provider. Second, the provider needed to view the app results before (or during) the time spent with the patient. Third, the ordering and administration of vaccines needed to occur following parents’ and providers’ app use. As such, we needed to identify a time during a visit when the parent could complete the intervention, a method for getting the completed intervention to the provider for review, and ensure that both steps took place before the time when vaccines need to be ordered during a clinical encounter. Thus, we selected the following discrete workflow components of interest: (1) patient locations, interactions, and tasks before meeting with the provider; (2) provider interactions with the patient and patient records; (3) the timing of vaccine record review; and (4) the timing of vaccine administration. We restricted our workflow components to activities that occurred outside the clinic exam rooms in order to preserve patient privacy, encourage patient participation, and follow the minimum necessary rule for data collection.

### Step 2: Workflow Assessment

To enhance the rigor of our direct observations, we created 2 standardized checklists by adapting the Arkansas Foundation for Medical Care Workflow Assessment Checklist found on the AHRQ website [34]. The first checklist (Multimedia Appendix 1) standardized recording observations of the patient from check-in to check-out and included fields about (1) patient locations, interactions, and tasks before meeting with the provider; (2) the timing of vaccine record review; and (3) the timing of vaccine delivery. The second checklist standardized the recording observations of the provider and included fields on provider interactions with patient records.

We created checklists in the MediDocs platform (EnMedical Systems), a software designed to capture medical information that includes a click to timestamp functionality. To ensure the usability and functionality of the Medidocs platform and enhance observer comfort with the procedures, observers created an example list of coffee shop procedures and then pilot tested the Medidocs platform at a local coffee shop by tracking employee and customer interactions. A coffee shop was selected because there were many individuals who followed the same procedures, and personal health information was not included.

We observed the clinical workflow at 4 primary care clinics that agreed to participate in the intervention implementation trial. The participating clinics were pediatric or family medicine specialties, served a range of patients with Medicaid insurance (14%-90% of patients), and were owned privately, by the university, or by health systems. Before the observation days, a lead clinical stakeholder at each clinic invited all medical staff who interacted with patients aged 11-12 years to attend an information session. Information sessions were held during each clinic’s preferred schedule (ie, during lunchtime or a staff meeting), food was provided, and research staff explained the study and informed consent forms. Clinic staff were given the opportunity to ask questions and return signed informed consent forms at any time before the observation days. The direct observation of clinical staff was restricted to those staff members who had returned signed consent forms.

To minimize clinic disruption and research staff travel expenses, we aimed to observe approximately 3 participants over 1-2 days at each clinic. Clinical staff selected days when at least one patient aged 11-12 years was scheduled. On each observation day, clinic staff invited all parents of patients aged 11-12 years to participate and referred interested parents to research staff who were present in the clinic. The research staff obtained consent through a Health Insurance Portability and Accountability Act waiver of documentation of consent and collected data without identifiers. Research staff conducting observations had a minimum of a bachelor’s degree in a scientific or social science field, had existing relationships with the clinic, and were trained in research and practice facilitation. We chose not to interview patients about the acceptability of completing the eHealth intervention during a clinic visit because parents were very receptive to completing the eHealth intervention during our pilot, and we enhanced parents’ acceptability of the app content with focus groups [6,42].

With 12 patients as a practical target, we observed 13 patient visits between August 2016 and March 2017. By the 13th visit, we were no longer observing new variations in the workflow components. Thus, saturation was reached, and further data collection was not needed [33]. However, we did continue to observe a variety of wait times but were able to conclude that sufficient time was typically available before the patient saw the provider.

### Step 3: Triangulation

For the triangulation of our direct observation, we interviewed clinical stakeholders (group interviews of a clinical support staff member and the lead physician at each clinic) because involving the clinic staff in the development process creates a sense of ownership and increases buy-in consistent with the CFIR [5,19,22]. We chose to visually present the workflow using a common flowcharting strategy [24]. The moderator was the same research staff who conducted direct observations. Each group followed a semistructured interview guide (Multimedia Appendix 2). To review the observed workflow, flowcharts of the acute and preventive care workflows were displayed on large poster boards. A moderator explained the flowchart, asked about any missing components, and solicited opinions on accuracy. Clinic staff were compensated US $20 for interview participation in approximately 20-minute sessions. Interviews were recorded and transcribed. The flowchart was updated for minor deviations. Evaluation activities used a postpositivist approach to observe and understand the main activities involved in clinic appointments.

**Figure 1:**
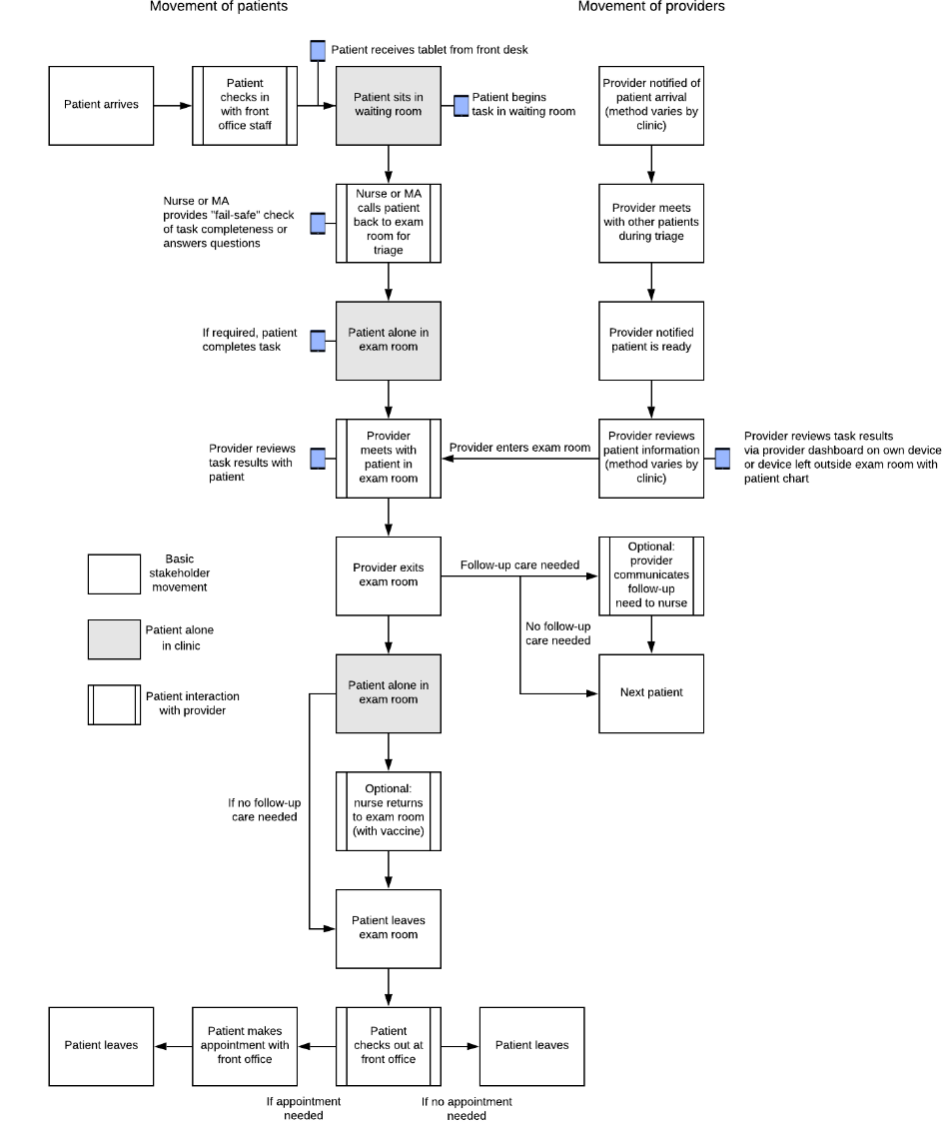
Example clinical workflow diagram of patient visits (those aged 11-12 years) with the proposed intervention tablet placement. MA: medical assistant.

After noting considerable similarities across clinics and visit types, we created a composite flowchart that represented the workflow at all 4 clinics (Figure 1). Our example flowchart shows that patients first arrived and were checked in with front office staff. Then, patients spent up to 59 minutes in the waiting room without staff contact (mean 12.6 min, SD 16.1 min in addition to time spent completing the study consent). In the 2 clinics, wait time was substantially less for acute than preventive visits. Next, a nurse or medical assistant triaged the patients’ presenting issue and collected basic vital information. Patients were then left alone in the exam room for a range of 2-25 minutes (mean 10.9 min, SD 8.8 min).

While a patient completed check-in and triage, physicians attended to other patients. Once notified that a patient was ready and waiting in an exam room, physicians reviewed the patient’s record for a few minutes immediately before entering the exam room. Depending on the clinic, patient records were reviewed via paper files placed immediately outside an exam room or electronically. Following the patient-physician interaction, physicians at each clinic communicated verbally with a nurse or medical assistant about additional care needs (eg, vaccine administration). The clinical staff administered ordered services. Finally, the patient returned to the front desk and scheduled another appointment or received the required instructions.

### Step 4: Stakeholder Proposal of Intervention Implementation

Clinicians suggested additional staff double-checking that the tablets were distributed. Participants mentioned that checking for tablet distribution during triage would be compatible with redundancy checks already taking place within a clinical workflow. “We always double-check ages and things anyway...so if I see that the patient is 11 or more, I can always grab [the tablet].” Another site noted that it would be helpful to use a team approach to enhance intervention dissemination. To accomplish this, front office staff could highlight the patients eligible for the intervention during their morning patient reviews. For example, one clinician stated:

They [front desk staff] identify all the 11- and 12-year-olds coming in for the following day, and they somehow highlight, red flag, that particular patient, and when we discuss patients coming in...it can just be mentioned. So, everyone, front desk staff, and clinicians, will know that a patient needs an iPad.

At all 3 of these clinics, physicians preferred to review the intervention results at the same time they reviewed the patient records. Clinicians suggested physically placing the tablet with the encounter forms and patient records or accessing the intervention results on their computer when reviewing patient records. As one physician explained, “Usually I look through the patient chart before I walk into the room, so I would just do that [review intervention results] right after.” In cases where patients were unable to complete the intervention in the waiting room, physicians felt that the parent could continue to complete the tablet while the physician interacted with the adolescent. In explaining this alternative, a physician noted:

...at age 11 or 12, I do more interaction with the child versus interaction with the parent. So, I can say, “Oh, by the way mom, can you go ahead and fill this out for me?”

One clinic that already used a tablet system suggested a slightly modified implementation. The staff of this clinic proposed that the new intervention be distributed in a similar fashion to their current tablet system (ie, given by nurse to parent in the exam room). If the parent finished before the nurse completed triage activities and left the exam room, then the nurse would collect the tablet from the parent and place it outside the exam room for the physician to review. If a parent was still completing the tablet intervention during the completion of triage activities, the nurse would alert the physician that the parent still had the tablet. In this case, physicians reviewed the tablet results upon entering the room alongside the patient. One physician explained:

I’d bring it up and say, “Let me see how you feel about the HPV vaccine,” and if they are ready to have it, “great,” and if not, I would discuss if they had a hesitation.

Clinicians identified 3 potential barriers to intervention implementation. First, clinicians reported that they do not typically review and administer vaccines during acute care visits. Although it was noted that parents would still be amenable to completing the intervention during most acute care visits, it was less clear how medical staff could alter their work processes to incorporate speaking about and, potentially, administering vaccines during acute visits. This issue was first mentioned in relation to the lack of an established process for checking vaccine records for patients presenting for an acute care visit, with staff reporting, “We don’t usually check ahead for the sick visit. Just for the wells.”

Second, physicians mentioned patient conditions that would preclude their administration of vaccines (eg, a high fever) or cause disruptions to how patients move through the clinic, thus interfering with when a tablet could be given to a patient to complete the intervention. One clinician explained that “...if we know that [a patient] is really, really contagious, we won’t even do two minutes [in the waiting room], as soon as they come in we put them in an empty room.” Clinicians were open to the idea of administering the intervention in these acute visit scenarios, for example, stating, “We could implement [the tablet] at some point, like once we’ve discussed the issue or whatever they’ve come in for,” but noting that more work would need to be done in order to optimize the process for acute visits.

The final barrier mentioned was the physician time constraints. One physician mentioned that, despite the simplicity of the tablet, competing demands may cause him to forgo reviewing intervention results stating*,* “If I’m in a rush, or I’m behind, or if we have lots of add-ons, what have you...I mean, it’s simple enough to just log-in and everything, but it’s that extra step.”

## Discussion

### Implications

We present a general strategy and an example for conducting a workflow assessment to enhance the implementation of an eHealth intervention in primary care. With this strategy, we were able to identify specific timing, staffing, and management processes that may enhance intervention implementation. Potential barriers related to the implementation of the intervention were identified and addressed by stakeholders. Incorporating clinical stakeholders into the design of the implementation plan enhanced stakeholder buy-in, a critical component of successful implementation of eHealth interventions [5]. By conducting the workflow analysis before the implementation of an eHealth intervention, researchers may avoid potential implementation barriers that limit the reach and effectiveness of the intervention.

Conducting the workflow assessment before intervention implementation allows for adaptations consistent with the adaptable periphery of interventions in the CFIR. By using a direct observation approach to assess clinical workflow, we were able to identify how and when our eHealth intervention should be incorporated. Clinicians’ participation in verifying the workflow and proposing an implementation strategy allowed for additional options to be proposed before implementation. For example, clinicians added a double-check of the tablet distribution and provided contingency plans for when parents did not have enough time to finish the app before seeing the provider. Although the clinicians were unaware that our prior pilot had issues with front office staff forgetting to give the tablets to parents, incorporation of clinicians in the planning process pre-emptively addressed this shortcoming in our second implementation. Moreover, while we had anticipated a workflow tailored to each clinic based on prior research [37], conducting the workflow assessment allowed us to simplify our approach with a standardized implementation plan that could be applied to all clinics with minimal tailoring.

By engaging stakeholders with implementation planning, we were able to identify potential barriers, discourage clinic staff adaptation of the core intervention, and build clinician buy-in. Consistent with previous primary care workflow research [37], we found that the clinical workflow differed between acute and well visits, suggesting that it will be more challenging to use the app during acute visits. However, clinicians suggested work-arounds for acute visits that were inconsistent with the core of the app. For example, parents using the app after their initial discussion with the provider allows clinicians to address immediate concerns but does not allow the app to aid parent-provider conversations. Learning of this potential work-around before implementation allowed research staff to explain the inconsistencies between the work-around and the core of the intervention and work with clinicians to develop alternative solutions (eg, limiting use of the app to nonurgent acute visits). Finally, and most importantly, through their involvement with the planning, clinicians gained a sense of ownership, which is expected to translate to a more successful implementation.

### Limitations

There are 3 important limitations of the case study presented that could be improved in future workflow assessments. First, the workflow assessment focused on physicians and patients. Although physician and patient visits are the most frequent opportunity for administering adolescent vaccines, a workflow assessment that included nurses and other clinical staff could be useful if the intervention was implemented in nurse-only visits. Second, our triangulation and stakeholder engagement strategies focused on physicians and clinical support staff. In particular, as front office staff were identified as integral to the implementation plan, including front office staff in the workflow assessment and intervention implementation planning would likely improve planning and garner the needed support from these critical stakeholders. Third, our case study presented 4 clinics with 3 observations each and, in turn, the small number of observations increases the risk of undue influence of outliers if the visits are not representative of standard care practices. However, this size is reasonable for a small implementation study and is supported by thematic saturation [43].

### Strengths

Our proposed workflow assessment strategy and supporting case study have 3 strengths relative to the design and execution. First, the use of 2 strategies to assess workflow enhances the rigor and validity of the results. Second, the use of separate observers for the patient and the physician was relatively novel and ensured that overlapping activities were observed. This strategy of one observer to each actor can be expanded to any clinical setting. Third, the benefit of involving the clinic staff in implementation planning with regard to stakeholder ownership and engagement cannot be understated. Participating clinicians are interested in seeing the intervention succeed and are aware that some work-arounds may alter the core of the intervention.

### Conclusions

Conducting a prospective workflow assessment can enhance the acceptability and performance of eHealth interventions in clinical practice. We presented a 4-step strategy to increase the compatibility between eHealth interventions and clinical workflow, as compatibility is one of the main predictors of clinician use [7,44]. Multiple options are presented for each of the 4 steps: (1) the identification of discrete workflow components, (2) workflow assessment, (3) triangulation, and (4) the stakeholder proposal for intervention implementation. By following the included step-by-step guide, eHealth researchers can study clinical workflows to design eHealth implementation strategies that complement rather than compete with clinical care. More importantly, through engagement of clinical staff in implementation planning, research staff can increase the clinical staff buy-in to the intervention and are able to stress that adaptations, while possible, need to be implemented only after considering the effects on the core intervention.

## Appendix

Multimedia Appendix 1 Direct observation checklist for patients and clinic staff. Boxes above and below the tables create timestamps upon clicking.

Multimedia Appendix 2 Semistructured interview questions and subquestions were used to evaluate workflow observations.

## Abbreviations

AHRQ: Agency for Healthcare Research and Quality
CFIR: Consolidated Framework for Implementation Research
EHR: electronic health record
HPV: human papillomavirus
