# Supplementary material for: Using a Clinical Workflow Analysis to Enhance eHealth Implementation Planning: Tutorial and Case Study
Source: JMIR Mhealth Uhealth. 2021 Mar 31;9(3):e18534. doi: 10.2196/18534 (PMC8047797; doi:10.2196/18534)
Supplement: Multimedia Appendix 1 [file mhealth_v9i3e18534_app1.pdf]

Patient tracker

Use the form below to track patient and nurse specific clinic workflow from patient check-in to check-out. Keep track of total time (beginning with patient entering the clinic) and time until completion of each task. Answer every question listed on the sheet and record details using the notes section to the right of each question.

Clinic and observer information

First name:	
Last name:	
Date:	
Clinic location:	
Observation start	

Observation begins following consent:

Check-In/Front Waiting Room	Notes
1. Approximately how long did you spend consenting?	
2. Is the parent required to complete additional paperwork after check-in? If so, in what format is the information collected?<br>☐ Electronic<br>☐ Paper<br>☐ Other	
a. How are nurses and providers notified of a patient's arrival?	
b. If the patient was asked to complete additional paperwork, how much time was spent filling out forms?	
c. Once completed, who did the patient give the paperwork to?	
d. Was the patient able to complete the paperwork before being called for their appointment?	
e. Who calls the patient from the waiting room?	
Time patient called back for appointment	

Pre-rooming activities start

Pre-rooming activities (patient triaging)	Notes
3. Are any activities conducted with the patient before rooming?<br>☐ Yes<br>☐ No<br><br>If yes, who conducts these activities? (Select all that apply)?<br>☐ Clinic staff (non-medical)<br>☐ Nurse (including staff like LPNs and PAs)<br>☐ Provider<br>☐ Other	
4. If observable, is the patient's immunization history reviewed?	
5. Is any paperwork collected from the patient during pre-rooming?	
6. How is pre-rooming information shared with providers?	
7. Who takes the patient to the exam room?<br>☐ Clinic staff (non-medical)<br>☐ Nurse (including staff like LPNs and PAs)<br>☐ Provider<br>☐ Other	

Patient enters exam room

Patient left alone in exam room

Rooming the patient and appointment conclusion	Notes
8. Once a patient is roomed, if observable, do their records continue to be reviewed?<br>☐ Yes<br>☐ No<br>If yes, who conducts these activities? (Select all that apply)?<br>☐ Clinic staff (non-medical)<br>☐ Nurse (including staff like LPNs and PAs)<br>☐ Provider<br>☐ Other ______	
9. Is patient immunization history reviewed or vaccines prepped following rooming the patient?<br>☐ Yes<br>☐ No<br>If yes, who conducts these activities? (Select all that apply)?<br>☐ Clinic staff (non-medical)<br>☐ Nurse (including staff like LPNs and PAs)<br>☐ Provider<br>☐ Other ______	
10. Do nurses and providers verbally discuss patient information after rooming and before the doctor sees the patient?<br>☐ Yes<br>☐ No	
11. How does the provider know the patient is ready to be seen?	
12. Does the provider bring materials (such as educational brochure) with them into the exam room?	
13. Time provider enters exam room<br>______<br>Time provider leaves exam room<br>______	
14. Does anyone return to the exam room with the patient after the provider leaves?<br>☐ Yes<br>☐ No<br>If yes, who conducts these activities? (Select all that apply)<br>☐ Clinic staff (non-medical)<br>☐ Nurse (including staff like LPNs and PAs)<br>☐ Provider<br>☐ Other ______	

Patient leaves exam room

	Patient returns to front office

Check-out	Notes
15. Does anyone escort the patient from the exam room to check out?<br>☐ Yes<br>☐ No<br>If yes, who conducts these activities? (Select all that apply)<br>☐ Clinic staff<br>☐ Nurse (including nursing staff)<br>☐ Provider<br>☐ Other ______	
16. Who conducts patient check-out?	
17. Does the patient bring information to check-out?	
18. Is a follow-up appointment scheduled?	
19. Is material/information given to the patient at check-out?	

Patient leaves clinic
