# Supplementary material for: Using a Clinical Workflow Analysis to Enhance eHealth Implementation Planning: Tutorial and Case Study
Source: JMIR Mhealth Uhealth. 2021 Mar 31;9(3):e18534. doi: 10.2196/18534 (PMC8047797; doi:10.2196/18534)
Supplement: Multimedia Appendix 2 [file mhealth_v9i3e18534_app2.pdf]

<table>
<tr><td>QUESTIONS</td><td>SUB-QUESTIONS/PROMPTS</td></tr>
<tr><td colspan="2">Workflow Diagram (show and describe diagram to participants)</td></tr>
<tr><td>1. How does the diagram match your experience with 11- to 12-year-old visits at the clinic?
2. Do you have any other suggestions about how to make the workflow diagram most reflective of your clinic?</td><td>• What revisions are needed for the diagram?
• What steps are missing from the diagram?
• What instances does your workflow differ from the picture?
  o For sick visits?
  o What about if running behind?
• Where do patients spend the most time waiting to see the doctor?
• How do the nurses and providers learn that a patient has arrived?
• How does patient information get handed between nurse and provider?
• Are patient vaccine needs identified for every 11- and 12-year-old patient, regardless of presenting issue?
• When do patients schedule follow up visits?</td></tr>
<tr><td colspan="2">Intervention Dissemination (describe basic eHealth intervention functionality)</td></tr>
<tr><td>1. Given the intervention description, at what point do you think would be the best time for the eHealth system to be given to the patient?
2. Where is the best location in the clinic for the parents to complete the eHealth intervention?</td><td>• Which clinic staff should give the iPad to the parents?
  o How does this person become aware that a patient has arrived?
• What would happen if this person was absent?
• Who do you think should handle questions about using the iPad and intervention?
• Who would be responsible for keeping the iPads maintained (e.g. charged and secure)?
• Where would the iPads be stored to make them easiest to access and maintain?</td></tr>
<tr><td colspan="2">Intervention Completion</td></tr>
<tr><td>1. Who should parents give the iPad to once they have finished?</td><td>• Where in the clinic should this exchange occur?
• How will the identified staff be alerted of whether the parent used the iPad?
• Where would the provider prefer to review the patient information entered into the iPad?
  o e.g., on own system, on tablet, etc.
• When would the provider be able to review the iPad report?</td></tr>
<tr><td colspan="2">Post Intervention Tasks</td></tr>
<tr><td>1. How should the iPad be taken back to make it available for the next patient?
2. Do you have any other suggestions about how the iPad system should be incorporated into the clinic workflow?
3. Is there anything else you would like to tell us related to the questions we have been discussion?</td><td>• Who will take the iPad from the clinician and return it to the individual responsible for handing out the intervention?
• Only parents of 11- to 12- year-old children are eligible to use the iPad system. How many iPads would you need to use to ensure adequate coverage?</td></tr>
</table>
